# Evolution of Bacterial Phosphoglycerate Mutases: Non-Homologous Isofunctional Enzymes Undergoing Gene Losses, Gains and Lateral Transfers

**DOI:** 10.1371/journal.pone.0013576

**Published:** 2010-10-26

**Authors:** Jeremy M. Foster, Paul J. Davis, Sylvine Raverdy, Marion H. Sibley, Elisabeth A. Raleigh, Sanjay Kumar, Clotilde K. S. Carlow

**Affiliations:** Division of Parasitology, New England Biolabs, Inc., Ipswich, Massachusetts, United States of America; University of Hyderabad, India

## Abstract

**Background:**

The glycolytic phosphoglycerate mutases exist as non-homologous isofunctional enzymes (NISE) having independent evolutionary origins and no similarity in primary sequence, 3D structure, or catalytic mechanism. Cofactor-dependent PGM (dPGM) requires 2,3-bisphosphoglycerate for activity; cofactor-independent PGM (iPGM) does not. The PGM profile of any given bacterium is unpredictable and some organisms such as *Escherichia coli* encode both forms.

**Methods/Principal Findings:**

To examine the distribution of PGM NISE throughout the Bacteria, and gain insight into the evolutionary processes that shape their phyletic profiles, we searched bacterial genome sequences for the presence of dPGM and iPGM. Both forms exhibited patchy distributions throughout the bacterial domain. Species within the same genus, or even strains of the same species, frequently differ in their PGM repertoire. The distribution is further complicated by the common occurrence of dPGM paralogs, while iPGM paralogs are rare. Larger genomes are more likely to accommodate PGM paralogs or both NISE forms. Lateral gene transfers have shaped the PGM profiles with intradomain and interdomain transfers apparent. Archaeal-type iPGM was identified in many bacteria, often as the sole PGM. To address the function of PGM NISE in an organism encoding both forms, we analyzed recombinant enzymes from *E. coli*. Both NISE were active mutases, but the specific activity of dPGM greatly exceeded that of iPGM, which showed highest activity in the presence of manganese. We created PGM null mutants in *E. coli* and discovered the *ΔdPGM* mutant grew slowly due to a delay in exiting stationary phase. Overexpression of dPGM or iPGM overcame this defect.

**Conclusions/Significance:**

Our biochemical and genetic analyses in *E. coli* firmly establish dPGM and iPGM as NISE. Metabolic redundancy is indicated since only larger genomes encode both forms. Non-orthologous gene displacement can fully account for the non-uniform PGM distribution we report across the bacterial domain.

## Introduction

Non-homologous ISofunctional Enzymes (NISE) is the preferred term to accurately describe enzymes that lack detectable sequence similarity but catalyze the same biochemical reactions and carry the same Enzyme Classification (EC) number [1]. NISE have previously been referred to as analogous enzymes [2], [3]. In many cases, NISE also lack structural similarity, this being a more robust indicator of independent evolutionary routes towards fulfilling a common metabolic conversion [3]. NISE most likely evolve by recruitment of existing enzymes that take on a new cellular function following changes to the substrate binding site or catalytic mechanism. This scenario is most plausible when one or both members of a pair of NISE belong to a larger enzyme family that catalyzes related reactions. For example, gluconate kinase from *Bacillus subtilis* has orthologs within the genus *Bacillus* but is otherwise unrelated to gluconate kinases from other bacteria or eukaryotes. However, the *Bacillus* enzyme belongs to a larger kinase family that includes xylulose kinase and glycerol kinase in other taxa. A duplication in the gene encoding either xylulose kinase or glycerol kinase is presumed to have occurred in the lineage leading to the Bacilli and been followed by a shift in substrate specificity to generate the novel gluconate kinase [3], [4]. Lateral gene transfer (LGT) events can further shape the distribution of NISE in different taxonomic groups and introduce enzyme activities analogous to ones already encoded by the recipient genome. The protozoan parasite, *Trichomonas vaginalis*, for example, encodes distinct forms of malic enzymes, one of which appears to be the result of LGT from a eubacterium [5]. The combination of enzyme recruitments and LGTs coupled with independent gene losses and gene gains in different lineages can therefore lead to patchy distributions of NISE forms when viewed across broad phylogenetic distances.

Phosphoglycerate mutase (PGM; E.C. 5.4.2.1.) catalyzes the interconversion of 2- and 3-phosphoglycerate (2-PG and 3-PG) in the glycolytic and gluconeogenic pathways. Two distinct forms of PGM that have no similarity in protein size, primary sequence, three-dimensional structure or catalytic mechanism are known to exist and are considered analogous enzymes (NISE) [1], [3], [6]. One form, cofactor-dependent PGM (dPGM), requires the cofactor 2,3-bisphosphoglycerate (2,3-BPG) for activity. The dPGM enzymes, having a molecular mass of about 27 kD, are usually active as dimers or tetramers and catalyze the intermolecular transfer of a phosphoryl group between the monophosphoglycerates and the cofactor via a phosphohistidine intermediate. Sequence and structural analyses of dPGM enzymes place them in the acid phosphatase superfamily along with enzymes such as fructose-2,6-bisphosphatase and acid phosphatase [7], [8]. On the other hand, cofactor-independent PGM (iPGM) is typically about 57 kD, active as a monomer, and catalyzes the intramolecular transfer of the phosphoryl group between monophosphoglycerates through a phosphoserine intermediate. The iPGM enzymes belong to the alkaline phosphatase superfamily along with enzymes such as phosphopentomutases and certain sulfatases to name a few [7], [8], [9]. The two forms of PGM can be distinguished further by the metal ion requirement of iPGM and the sensitivity of dPGM to vanadate [8], [10].

PGM sequences, in particular those of iPGM, appear to be evolving very slowly [7] and are generally very well conserved even across different kingdoms [8], allowing their identification in genome sequences from diverse organisms. However, since both dPGM and iPGM are members of larger phosphatase superfamilies containing diverse enzymes with related sequences, the identification of PGMs solely by sequence similarity should be treated with caution. Indeed, a predicted dPGM of *Bacillus* spp. was subsequently shown by molecular modeling and enzymatic analyses of recombinant protein to encode a broad specificity phosphatase [11]. Small-scale bioinformatic surveys and biochemical studies have indicated that only iPGM is present in plants and nematodes while only dPGM is found in mammals [6], [10], [12], [13]. However, within other phylogenetic groups the distribution of the two PGM forms is complex and has been described as appearing haphazard [6]. Most bacteria, archaea, protozoa and fungi contain either iPGM or dPGM, while some bacteria such as *Escherichia coli* and certain archaea and protozoa contain both forms. The respective roles of dPGM and iPGM in organisms that contain both forms of enzyme are uncertain.

In *E. coli,* at least, distinct PGM activities were reported for both dPGM and iPGM in crude cell extracts and when expressed in recombinant form [6]. The dPGM form accounted for the great majority of activity leaving unanswered questions about the role of iPGM in *E. coli*. To gain insight into the respective functions of dPGM and iPGM in *E.coli*, we generated null mutants for phenotypic studies to examine the role of each enzyme. We report that loss of dPGM leads to delayed growth both in liquid cultures and on solid medium, apparently due to a delay or defect in exiting stationary phase. We further show that the wild type phenotype can be restored by overexpression of either dPGM or iPGM in *dPGM* null mutants. We also produced recombinant dPGM and iPGM for detailed biochemical analyses to address the specific PGM and phosphatase activities of each enzyme. We demonstrate that the distinct PGM forms present in *E.coli* have overlapping and complementary roles in the cell.

The evolutionary origins of dPGM and iPGM that underlie the unpredictable distribution of these NISE proteins in bacteria are not clear [7], [8]. However, the abundance of sequenced microbial genomes provides an unprecedented opportunity to address the distribution of NISE across hundreds of bacterial species. In the present study we performed a comprehensive survey of the distribution of the PGM forms throughout the bacterial domain to gain insight into the processes and events that appear to have contributed to their apparently haphazard phyletic profiles.

## Materials and Methods

### Bioinformatic identification of iPGM and dPGM in bacterial genomes

The 702 completed microbial genomes listed in Table S1 were downloaded from NCBI Refseq (ftp://ftp.ncbi.nih.gov/genomes/Bacteria/) on October 18^th^, 2008.

A set of proteins was compiled which encompassed examples of divergent bacterial dPGM and iPGM proteins, archaeal iPGM and dPGM, as well as closely related, but functionally divergent, acid and alkaline phosphatase superfamily members that could complicate bioinformatic identification of bacterial PGM by generating false positives. Using TBLASTN [14], these query proteins (*E. coli* GpmA (dPGM), NCBI GI number 50402115; *Chlamydia trachomatis* dPGM, 15605455; *E. coli* GpmM (iPGM), 586733; *Ureaplasma parvum* iPGM, 13357740; *Thermoplasma acidophilum* (archaeon) dPGM [15], 10640690; *Pyrococcus furiosus* (archaeon) iPGM [16], 18894161; *E. coli* gpmB (dPGM family member), 67465002; *Bacillus subtilis* phosphatase, PhoE, 2633370; *Mycobacterium tuberculosis* phosphatase [17], 38490339; *E. coli* phosphopentomutase, DeoB, 170083769 and *E. coli* alkaline phosphatase, PhoA, 48994877) were aligned against the six-frame translations of the set of completed microbial genomes. References to publications that establish the function of the above query proteins are provided in those instances where original NCBI functional definition of the query proteins was either lacking or incorrect. TBLASTN was provided a value of 10,000 for both the one-line descriptions and alignments parameters and a value of 10 for E-Value cutoff, with all other parameters left at default values. All TBLASTN alignments with a bit score less than 100.0 were discarded. The bit score cutoff of 100 was established empirically by examination of the output produced using a range of bit score cutoffs (data not shown).

Using the alignments passing the bit score threshold, a list of automatic PGM assignments was generated for each genome using the pattern of TBLASTN hits for the query proteins as follows. If the genome had hits with overlapping genomic coordinates for the dPGM queries from *E. coli* and *C. trachomatis*, it was automatically called as “dPGM”. Similarly, if the genome had overlapping hits for the iPGM queries from *E. coli* and *U. parvum* it was called as “iPGM”. If the genome had hits for the two dPGM and two iPGM queries it was called as “iPGM plus dPGM” (both forms).

The genome coordinates of additional hits arising from any of the 7 other queries were examined to identify instances where the protein aligned to the same genomic locus as PGM or one of the other query proteins. This step served to highlight any cases where sequence similarity searches failed to differentiate between either of the PGM forms and functionally diverged proteins from the same phosphatase superfamily. Genomes for which no assignment was automatically made usually contained more than one copy of a given PGM type, or PGM similar to an archaeal PGM query, or lacked any form of PGM. Such cases were curated following manual inspection.

To verify the orthology assignments determined by TBLASTN, we recovered each identified gene and used BLASTP (default arguments) against the *E. coli* MG1655 genome (GI: 49175990) to check that the corresponding PGM form in *E. coli* MG1655 (iPGM GI:16131483, dPGM GI:16128723) was the top ranking hit. In all cases except one, this check was successful. The single exception was a dPGM gene from *Akkermansia muciniphila* (GI: 187735276) that apparently has a full-length dehydrogenase gene (encodes ∼330 amino acids) fused to the 3′ end of a predicted PGM gene. In this case, the *E. coli* dPGM was the second best hit while the top hit was to the orthologous dehydrogenase.

The taxonomic designations of all organisms described in this study are consistent with the NCBI Taxonomy Browser. In the Results section (Tables and Figures), PGM distribution data is generally presented at the Class taxon so as to adequately reveal the non-uniform nature of PGM while limiting the number of bacterial genomes displayed.

### Mapping the likely origin of archaeal PGM genes in bacterial genomes

The output from the TBLASTN analysis was also used to find genes in bacterial genomes that contained hits to the archaeal iPGM or dPGM queries with a bit score exceeding 100. The archaeal-like genes were then used as queries against all completely sequenced archaeal genomes downloaded from NCBI (ftp://ftp.ncbi.nih.gov/genomes/Bacteria/) on October 18th, 2008 using the same TBLASTN parameters as described above. The score for each archaeal species was then calculated as the average bit score of the best blast hits for all PGM query sequences. Where multiple genome sequences for one species are available, only the single top bit score from across all sequences was used in the calculation.

### Bacterial strains, media and growth

Recombinant GpmA (dPGM) and GpmM (iPGM) were expressed in *E. coli* T7 Express (New England Biolabs). Deletions of *gpmA* (*dPGM*) or *gpmM* (*iPGM*) were made in the *E. coli* K-12 derivative, MG1655 (*E. coli* Genetic Stock Center). Bacteria were grown in Luria Bertani (LB) medium (10 g tryptone, 5 g yeast extract, 5 g NaCl per liter H_2_0, pH 7.2) and in 3-(N-morpholino)propanesulfonic acid (MOPS) minimal medium [18] (TekNova), supplemented with 0.1% glucose. For production of recombinant proteins or complementation by plasmid constructs, ampicillin (100 µg/ml) was included in the growth medium. All bacterial growth was at 37°C and liquid cultures were shaken at 250 r.p.m.

### PGM cloning, expression and enzyme assays

Full-length *E. coli iPGM* and *dPGM* were cloned into the pET-21a vector (Novagen) for expression of recombinant proteins with C-terminal His_6_ tags in *E. coli*. The sequences were amplified (see Table S2 for primers) from genomic DNA from *E. coli* strain T7 Express using the Expand High Fidelity PCR System (Roche). Constructs were verified by DNA sequencing before expression of the recombinant proteins in T7 Express *E. coli*. Optimal expression of both iPGM and dPGM was achieved following induction with 0.3 mM isopropyl-1-thio-β-D-galactopyranoside (Sigma) for 3 h at 37°C. The His-tagged proteins were extracted and purified on nickel columns (Qiagen) under native conditions according to the manufacturer's instructions.

Purified recombinant proteins were assayed for PGM activity in the glycolytic direction (3-PG to 2-PG) using a standard enzyme-coupled assay as described previously [19]. Briefly, PGM was added to 1 ml assay buffer (30 mM Tris-HCl, pH 7.0, 5 mM MgSO_4_, 20 mM KCl) supplemented with 0.15 mM NADH, 1 mM ADP, 1.5 mM 3-PG substrate (Sigma P8877) and 2.5 units of each coupling enzyme, namely enolase (Sigma E6126), pyruvate kinase (Sigma P7768) and L-lactic dehydrogenase (Sigma L2518). Reactions were at 30°C for 5 min with data collected every 10 s using a DU 640 spectrophotometer (Beckman). Consumption of NADH at 340 nm provided an indirect measurement of PGM activity as the amount of NADH converted to NAD corresponds to the amount of reaction product, 2-PG. One unit of PGM activity is defined as the amount of activity necessary to convert 1.0 µmole NADH to NAD per minute under the standard assay conditions. The effect of manganese ions was studied by adding manganese chloride to the standard assay buffer to a final concentration of 1 mM. Sensitivity to vanadate was addressed by incubating the recombinant enzymes with different concentrations of sodium metavanadate (Acros) for 15 min. prior to the assay. The activity of dPGM was determined in the absence of the cofactor, 2,3-BPG, since the commercially available 3-PG substrate for PGM assays contains 2,3-BPG as a contaminant in sufficient amounts to stimulate dPGM activity causing an apparent lack of dependency on cofactor [15].

Phosphatase activity was assessed in 200 µl reactions using 10 µg enzyme and 50 mM *p*-nitrophenyl phosphate (New England Biolabs) as substrate. Various buffer systems were used: NEBuffer 3, pH 7.9, NEBuffer EcoRI, pH 7.5 (both from New England Biolabs), PGM assay buffer, pH 7.0 (see above), and 1 M diethanolamine, 1 mM MgCl_2_ **,** pH 9.75. The effect of different metal ions was determined by addition of either ZnCl_2_ or CoCl_2_ to these four magnesium-containing buffers. Calf intestinal phosphatase (New England Biolabs) served as an alkaline phosphatase positive control in each buffer. Reactions were incubated at 37°C for 30 min before being stopped by addition of 1 ml 1N NaOH. The production of *p*-nitrophenylate was determined spectrophotometrically at 405 nm and compared to controls lacking either substrate or enzyme.

### Construction and characterization of *E. coli* PGM mutant strains

Separate strains bearing either a deletion of the entire *iPGM* or *dPGM* open reading frame of *E. coli* MG1655 were prepared by λ Red-mediated recombination [20]. PCR primer pairs were designed (Table S2) such that their 5′ ends corresponded to the sequence immediately upstream and downstream of each PGM translational start and stop codon, respectively, while the 3′ ends of each primer pair corresponded to the P1 and P2 priming sites of the pKD4 plasmid [20]. The gene deletions in the resultant strains, MG1655*ΔgpmM::FRT1* and MG1655*ΔgpmA::FRT1* (*ΔiPGM* and *ΔdPGM*, respectively), were confirmed by PCR with diagnostic primers and by DNA sequencing. FRT1 indicates a FLP recombinase recognition site left at each locus after removal of a kanamycin cassette used during strain construction [20].

The growth of the *ΔdPGM* and *ΔiPGM* strains relative to the MG1655 parental strain was assessed by diluting overnight cultures grown in MOPS minimal medium supplemented with 0.1% glucose into 10 ml fresh LB medium in Nephelo sidearm flasks (Bellco Biotechnology) to give initial OD_600_ values of 0.03. Each strain was grown in triplicate and turbidity monitored using a photoelectric colorimeter (Klett Summerson**).** For evaluating growth on solid media, overnight cultures grown in MOPS minimal medium containing 0.1% glucose were standardized to similar optical density, when necessary, then serially diluted and 100 µl of each dilution plated in triplicate to LB agar. The number of colonies on each plate was recorded after overnight growth.

### Complementation of *ΔdPGM*

To examine whether *E. coli iPGM* or *dPGM* could complement the *ΔdPGM* growth phenotype, these genes were cloned into the pKK223-3 expression vector (Amersham Pharmacia Biotech) and transformed into the *ΔdPGM* mutant strain. The sequences were amplified (see Table S2 for primers) from the pET-21a constructs described above using Phusion High Fidelity DNA polymerase (New England Biolabs), then cloned into pKK223-3 and verified by DNA sequencing. The constructs were designated pKK*iPGM* and pKK*dPGM*. For complementation assays, strains MG1655, *ΔdPGM*, and *ΔdPGM* harboring, separately, pKK*iPGM* and pKK*dPGM* were initially grown overnight in MOPS minimal medium containing 0.1% glucose. These cultures were then serially diluted and plated in triplicate to LB agar as described above. Strain *ΔdPGM* harboring empty plasmid, designated pKK, served as a control.

## Results and Discussion

### Validation of the selected PGM superfamily members as queries for ortholog detection

The distribution of dPGM and iPGM was previously reported from small-scale bioinformatic and biochemical studies [6], [10], [12], [13]. Here we took advantage of the abundance of microbial genome sequences to comprehensively examine the distribution of the PGM NISE across 702 complete bacterial genomes (Table S1). We reasoned that use of divergent dPGM and divergent iPGM queries for our TBLASTN analyses would maximize identification of their bacterial orthologs and reduce/eliminate false negatives. We also used a variety of functionally divergent protein queries from the acid and alkaline phosphatase superfamilies to which dPGM and iPGM respectively belong. Since in most cases these superfamily members show sequence similarity to PGM, careful analysis of their BLAST hits was necessary to reduce/eliminate false positive identification of PGM.

#### PGM query proteins

For identification of dPGM orthologs in bacterial genomes by TBLASTN analysis, we selected the experimentally validated *E. coli* dPGM (GpmA) [6] and dPGM from *C. trachomatis* as queries. The latter dPGM shows considerable divergence from the *E. coli* ortholog, but passed our 100 bit score threshold for assignment as a PGM. The two proteins give reciprocal best BLAST hits between their genomes establishing their orthology. The *C. trachomatis* dPGM, although not experimentally validated, has higher similarity to the biochemically characterized dPGM from *Schizosaccharomyces pombe* than it does to *E. coli* dPGM. The *C. trachomatis* protein lacks a stretch of ∼25 amino acids when compared to dPGM from *E. coli* and most other organisms. Although this missing loop region is the least conserved region of dPGMs [11], [21], it contains amino acids important for dimerization or tetramerization [22]. Interestingly, *S. pombe* dPGM, which has been characterized in detail, also lacks this region and is active as a monomer [23], [24], suggesting that certain bacterial dPGM forms, such as that from *C. trachomatis*, are also monomeric. We noted that this type of dPGM, lacking the dimerization/tetramerization domain, is common within the order Chlamydiales and phylum Cyanobacteria (orders Chroococcales and Gloeobacteria), as well as the order Rhizobiales (α-proteobacteria). It was also observed in *Pseudoalteromonas atlantica* (γ-proteobacteria), *Myxococcus xanthus* (δ-proteobacteria) and *Sulfurihydrogenibium* sp. (Aquificales). However, we found that members of the order Chlamydia and the cyanobacteria that lack this region of dPGM generally have an insertion of ∼25 amino acids nearer to the N-terminus. The significance, if any, of this region is unknown. Despite the use of divergent dPGM proteins for our TBLASTN analysis, we determined that the two queries always generated the same hits (overlapping genome coordinates) on the bacterial genomes. To identify iPGM orthologs in the bacterial genomes we selected the experimentally characterized iPGM from *E. coli* (GpmM) [6] and the divergent *U. parvum* iPGM as queries for our TBLASTN analyses. These query proteins were also established as orthologs via reciprocal best BLAST hits. These two iPGM queries also always showed overlapping hits on the bacterial genomes. These observations are in agreement with the known well-conserved nature of the two PGM forms across different taxa and provides confidence that we identified all (or most) of the bacterial enzymes. The dPGM and/or iPGM genes identified in each bacterial genome were verified as orthologs of the characterized *E. coli* PGM genes by returning as best BLAST hits the appropriate PGM gene of *E. coli*.

We also used the sequences of biochemically characterized dPGM and iPGM proteins from the archaea *T. acidophilum* and *P. furiosus*, respectively, to query the complete bacterial genome sequences. We did not detect any archaeal dPGM orthologs in the bacterial genomes but found several examples of archaeal iPGM. The loci of the archaeal-like iPGM sequences we identified in bacterial genomes were in all cases distinct from those revealed by the bacterial iPGM and dPGM queries, again indicating that our parameters were sufficiently sensitive to differentiate closely related sequences.

#### Alkaline and acid phosphatase superfamily members as query proteins

Although our divergent iPGM and dPGM query proteins gave clear and consistent results, it is known that identification of PGM orthologs based on sequence similarity alone can be unreliable because of their similarity to functionally more divergent members of the alkaline and acid phosphatase superfamilies to which they belong [11]. We addressed this possibility by including as queries for our TBLASTN analysis of the bacterial genomes, various superfamily member proteins, which could reveal false positive PGM identification or cases where functional assignment by sequence similarity is ambiguous. For this purpose we used well characterized proteins such as phoE, a broad-specificity phosphatase from *B. subtilis*, and a *Mycobacterium tuberculosis* phosphatase, both of which were originally annotated as dPGM [11], [17]. We included other representative superfamily members, namely deoB, an *E. coli* phosphopentomutase, and phoA, an *E. coli* alkaline phosphatase, which could also confound interpretation of the BLAST outputs [9], [25]. While these four additional queries returned hits from various genomes (Table S1), there was not a single instance where a hit with a BLAST bit score greater than 100 had overlapping genome coordinates with hits returned by the dPGM or iPGM queries. This indicates that a bit score threshold of 100 appears to reliably differentiate the various superfamily members. We did not use more distant superfamily members such as SixA phosphoprotein phosphatase and Ais as queries since these are known not to have significant match to dPGM in standard BLAST searches [26]. However, a second dPGM-like gene, phosphoglycerate mutase B (GpmB) has been noted previously in various Enterobacteriaceae [15]. We identified candidate orthologs of this protein not only in the γ-proteobacteria but in other diverse bacterial taxa (Table S1). Once again, the dPGM and GpmB hits on all such genomes were non-overlapping. In fact we noticed overlap of the hit coordinates for the GpmB and phoE queries on several occasions, particularly in the Enterobacteriales but also in the Clostridiales, suggesting that GpmB may be an acid phosphatase. These analyses increase our confidence that our identification of PGM orthologs was robust since they showed that the distribution and genomic loci of orthologs of known PGM superfamily members, or other sequences closely related to PGM, had no overlap with those of dPGM or iPGM.

### Overview of distribution of dPGM and iPGM across the bacterial domain

**Figure 1:**
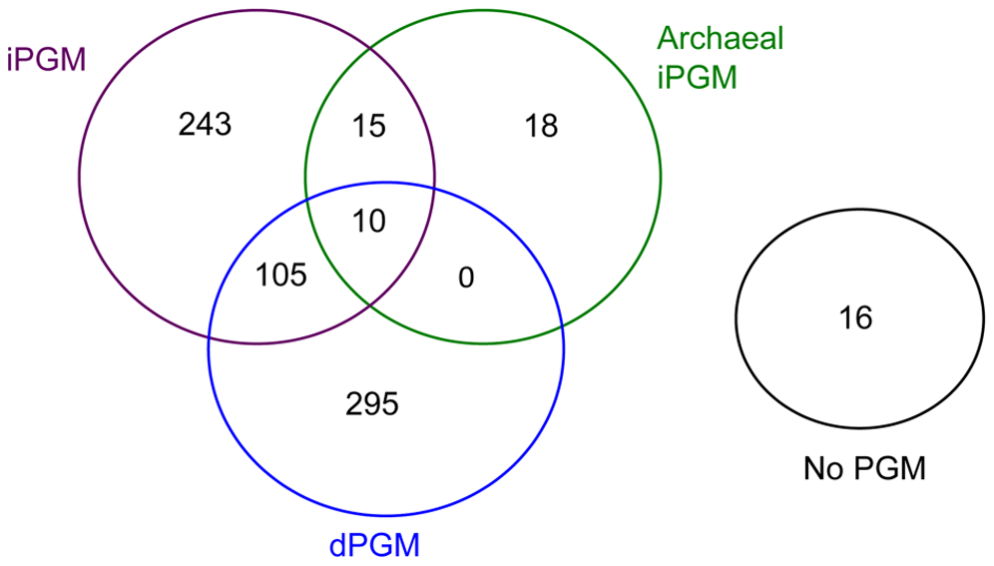
Distribution of dPGM, iPGM and orthologs of archaeal iPGM across 702 completed bacterial genome sequences.

After removal of duplicate genomic sequences for some bacterial strains, we calculated that the dPGM queries had 447 hits on 410 genomes (∼1.1 hits/genome) with a range of 0 to 3 hits per genome (Table S1). Thirty-four genomes had more than one dPGM. Of the 410 genomes containing dPGM, 115 also had at least one iPGM hit (Fig. 1). No eubacterial genomes had hits above the bit score threshold of 100 when the biochemically characterized dPGM from the archaeon *Thermoplasma acidophilum* [15] was used a query. There were 430 iPGM hits on 391 genomes (∼1.1 hits per genome) with a range of 0 to 4 hits per genome. However, only in 4 diverse bacteria (discussed below) was more than one iPGM identified by the two bacterial iPGM queries used. Considering only these “bacterial type” iPGMs, we report 380 hits on 373 genomes (∼1.0 hit/genome). The experimentally validated archaeal iPGM from *Pyrococcus furiosus* [16] identified 50 archaeal-like iPGM sequences in 43 bacterial genomes (Fig. 1), presumably as a result of LGT, thereby increasing the apparent frequency and number of iPGM hits per genome. The genome coordinates of the archaeal iPGM hits were distinct from those for the two bacterial iPGM queries in all cases. Of interest, eighteen bacterial genomes contained archaeal type iPGM as their only PGM form (Table 1; Fig. 1; Table S1).

**Table 1:** Summary of dPGM and iPGM distribution across different bacterial taxa.

Phylum	Taxon Group	Genomes	Total none	Total D+I	Total D	Total I	Total Archaeal I	Total Only Archaeal I
Proteobacteria	**Alphaproteobacteria**	89	13	3	43	30	0	0
	**Betaproteobacteria**	60	0	0	56	4	0	0
	**Gammaproteobacteria**	184	1	57	46	80	0	0
	**Deltaproteobacteria**	21	0	7	1	13	11	0
	**Epsilonproteobacteria**	21	0	1	1	19	0	0
Actinobacteria	**Actinobacteria**	53	0	1	51	1	1	1
Firmicutes	**Bacilli**	96	0	32	53	11	0	0
	**Clostridia**	37	0	5	2	30	7	0
Bacteroidetes	**Bacteroidetes**	8	0	5	2	1	5	0
	**Flavobacteria**	4	1	0	0	3	0	0
	**Sphingobacteria**	2	0	1	0	1	0	0
Chlorobi	**Chlorobia**	11	0	0	11	0	0	0
Fusobacteria	**Fusobacteria**	1	0	0	1	0	0	0
Thermotogae	**Thermotogae**	7	0	0	0	7	7	7
Chlamydiae	**Chlamydiae**	13	0	0	13	0	0	0
Verrucomicrobiae	**Verrucomicrobiae**	1	0	1	0	0	0	0
	**Opitutae**	1	0	0	0	1	0	0
Spirochaetes	**Spirochaetes**	16	0	0	10	6	0	0
Cyanobacteria	**Chroococcales**	15	0	1	0	14	0	0
	**Oscillatoriales**	1	0	0	0	1	0	0
	**Nostocales**	3	0	0	0	3	0	0
	**Prochlorales**	12	0	0	0	12	0	0
	**Gloeobacteria**	1	0	0	1	0	0	0
Acidobacteria	**Acidobacteria**	1	0	0	0	1	0	0
	**Solibacteres**	1	0	0	1	0	0	0
Aquificae	**Aquificae**	3	0	0	2	1	1	1
Chloroflexi	**Dehalococcoidetes**	3	0	0	0	3	3	3
	**Chloroflexi**	4	0	0	0	4	0	0
Plactomycetes	**Planctomycetacia**	1	0	0	0	1	1	0
Deinococcus-Thermus	**Deinococci**	4	0	0	0	4	4	4
Tenericutes	**Mollicutes**	22	1	0	0	21	0	0
Dictyoglomi	**Dictyoglomia**	1	0	0	0	1	1	1
Nitrospirae	**Nitrospira**	1	0	0	0	1	1	1
Unclassified	**Unclassified**	4	0	1	1	2	1	0

The number of genomes in each taxon identified as containing only iPGM, only dPGM, both iPGM and dPGM, and no PGM are given. The number of bacterial genomes containing archaeal type iPGM are given and are a subset of the total iPGM and/or total iPGM and dPGM categories. Genomes containing archaeal iPGM as their only PGM form are also enumerated. The taxonomic groupings shown in bold type are those used predominantly in this study and are taken from the NCBI Taxonomy Browser. All are classes except for the orders Chroococcales, Nostocales, Oscillatoriales and Prochlorales (from the phylum Cyanobacteria and lacking any class designation in the NCBI taxonomy database), and the phylum Bacteroidetes, which encompasses 7 genomes from the class Bacteroidia plus one incompletely classified Bacteroidete member. Four species with incomplete lineage designations are grouped at bottom of the table as “Unclassified”.

Sixteen genomes did not contain any form of PGM (Table 1; Fig.1). These organisms included the α-proteobacterial *Rickettsia* spp and closely related *Orientia* spp., together with Candidatus *Sulcia muelleri* (Flavobacterium), Candidatus *Carsonella ruddii* (γ-proteobacterium) and Candidatus *Phytoplasma mali* (Mollicute) (Table 1; Table S1). These are all intracellular bacteria with reduced genomes ranging from 2.1 Mb (*O. tsutsugamushi*) to the smallest known bacterial genome of 160 kb (Candidatus *Carsonella ruddii*) that lack all or part of the glycolytic pathway.

Examination of the presence of the PGM NISE across different bacterial taxa revealed a strikingly non-uniform distribution (Table 1) as noted previously [6]. This was generally most evident for taxa such as the α-, δ- and γ-proteobacteria, the Clostridia and the Bacilli which contain greater numbers of fully sequenced genomes. Other groups often contained very few sequenced genomes or a limited diversity of sequenced species thereby potentially masking PGM heterogeneity within those groups. For example, the 12 completed genomes within the order Prochlorales are from different strains of the same species. However, even different strains of *Prochlorococcus* [27] and other species [28], [29], [30] may have considerable variation in their gene content. In the case of *Frankia* spp., as many as 3,500 genes (∼50% of the predicted ORFs) may differ between strains [29], [31]. The non-uniform distribution of PGM NISE did not appear to correlate with any obvious trait such as aerobic/anaerobic metabolism, pathogenicity, or Gram staining.

### PGM Diversity within bacterial taxa

**Figure 2:**
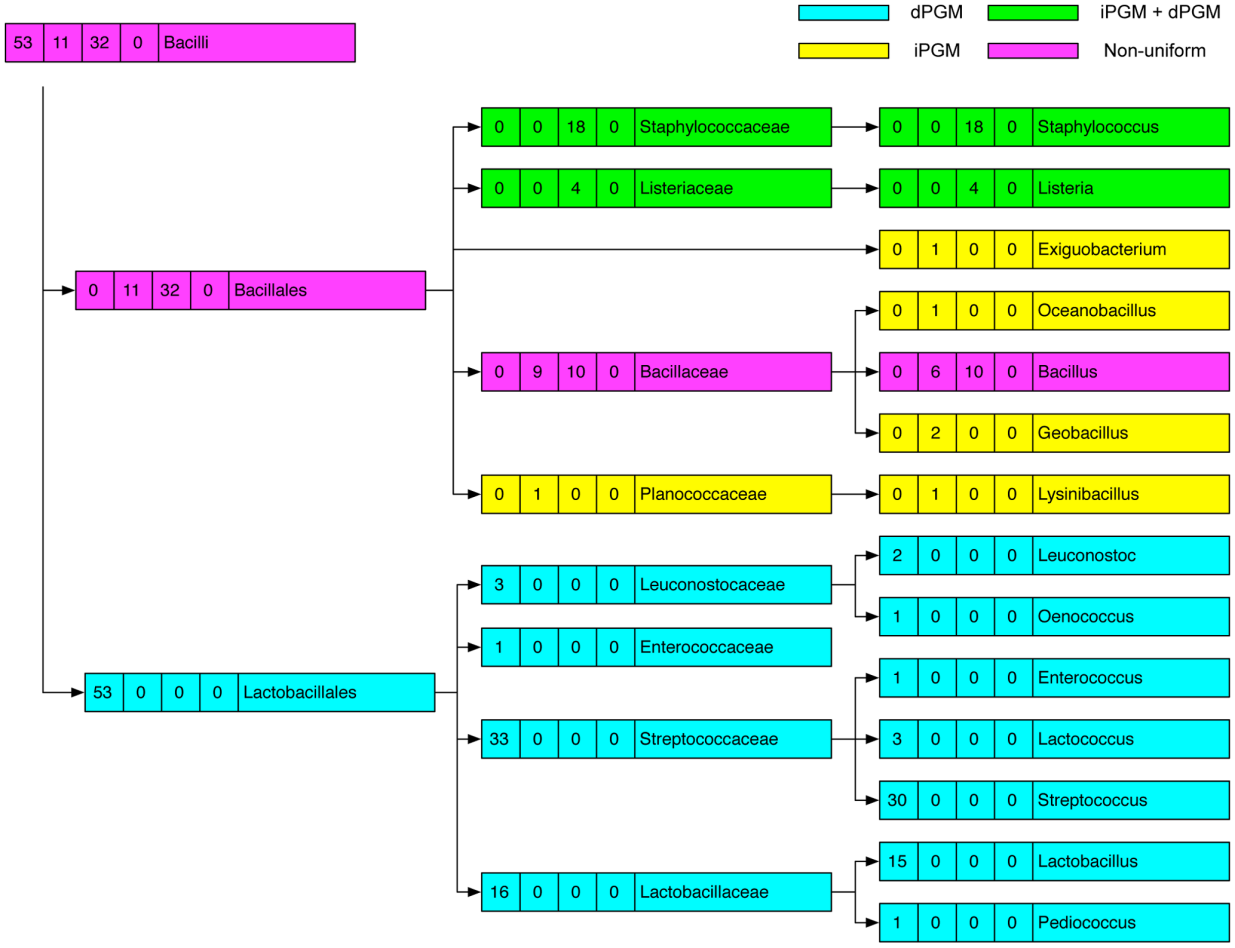
Distribution of PGM types across 96 completed genome sequences from the Class Bacilli. Taxonomic nodes (left to right) are Class, Order, Family, Genus. Taxa with genomes containing only iPGM are shaded yellow, those with only dPGM are shaded blue, those with both iPGM and dPGM are shaded green while taxa with non-uniform PGM profiles are shaded pink. The numbers in boxes accompanying each taxon identifier correspond to (left to right) number of genomes with only dPGM, only iPGM, both dPGM and iPGM, and no PGM.

**Figure 3:**
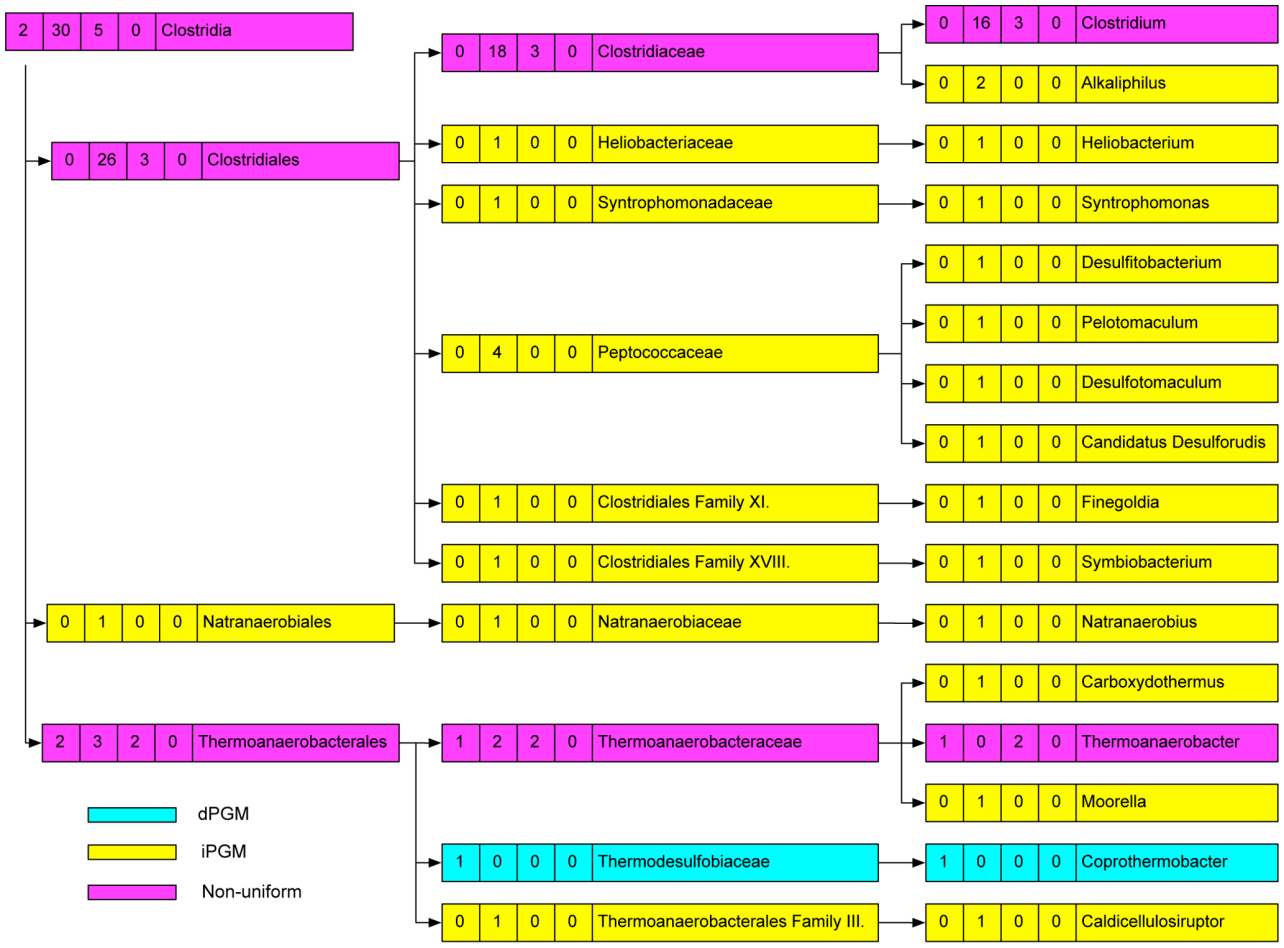
Distribution of PGM types across 37 completed genome sequences from the Class Clostridia. Taxonomic nodes (left to right) are Class, Order, Family, Genus. Taxa with genomes containing only iPGM are shaded yellow, those with only dPGM are shaded blue, those with both iPGM and dPGM are shaded green while taxa with non-uniform PGM profiles are shaded pink. The numbers in boxes accompanying each taxon identifier correspond to (left to right) number of genomes with only dPGM, only iPGM, both dPGM and iPGM, and no PGM.

**Figure 4:**
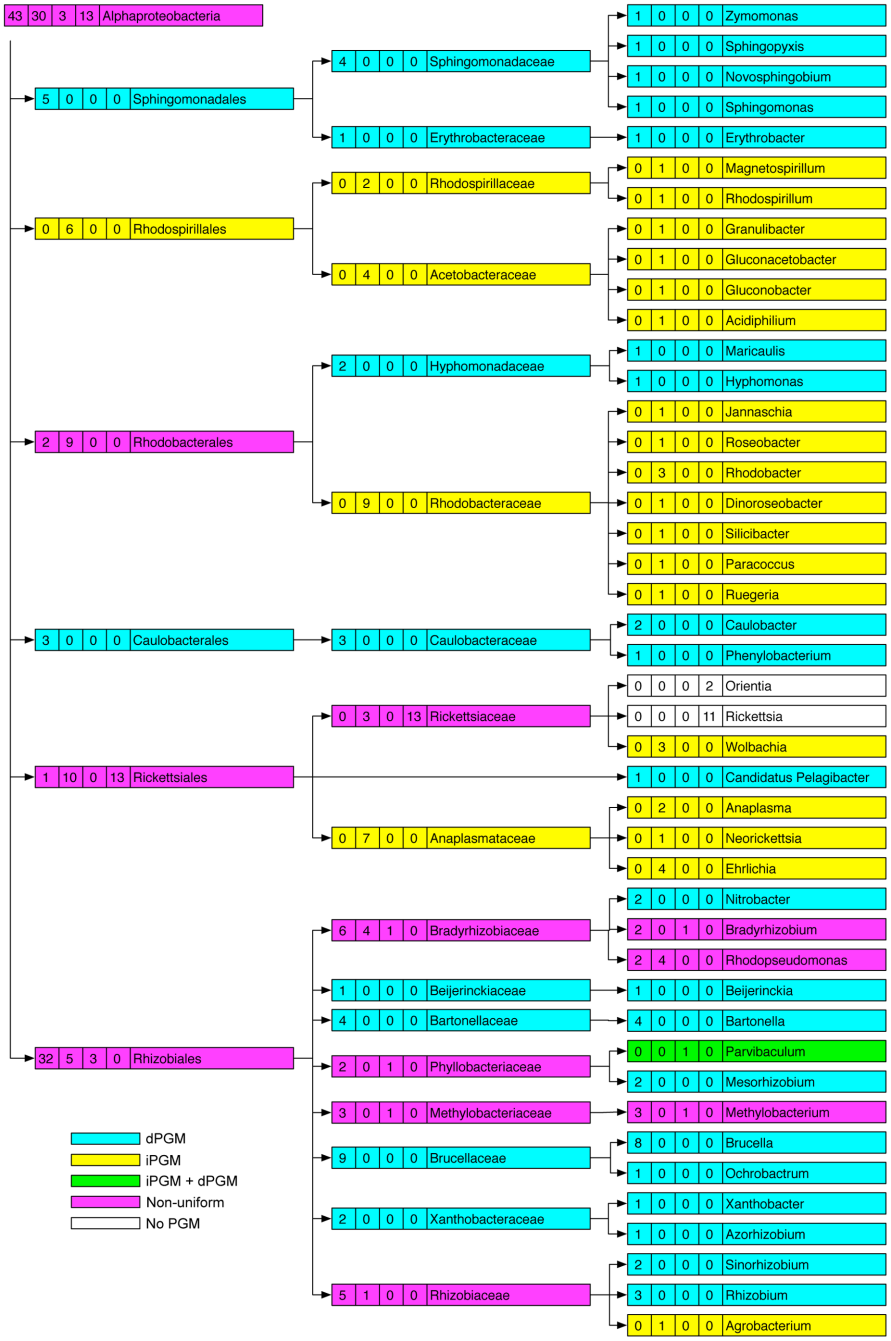
Distribution of PGM types across 89 completed genome sequences from the Class α-proteobactria. Taxonomic nodes (left to right) are Class, Order, Family, Genus. Taxa with genomes containing only iPGM are shaded yellow, those with only dPGM are shaded blue, those with both iPGM and dPGM are shaded green while taxa with non-uniform PGM profiles are shaded pink. Taxa with no PGM are unshaded. The numbers in boxes accompanying each taxon identifier correspond to (left to right) number of genomes with only dPGM, only iPGM, both dPGM and iPGM, and no PGM.

We found that much of the PGM heterogeneity observed in certain classes of bacteria (Table 1) stratified when individual families and genera were considered. For example, the diversity observed in the class Bacilli (Table 1) was resolved by examination of different families and genera (Fig. 2). Although a comparison between different families or genera revealed divergent PGM profiles, of 9 represented families, only the Bacillaceae exhibited diversity within its PGM profile, and of 13 genera, only the genus *Bacillus* (6 iPGM; 10 iPGM plus dPGM) had a non-uniform distribution (Fig. 2). Similarly, the 66 genomes from the family Enterobacteriaceae (γ-proteobacteria) (12 dPGM; 54 dPGM + iPGM) come from 17 genera, each of which is internally homogeneous: either a genus had exclusively dPGM or it had dPGM plus iPGM (Fig. S1). Nonetheless, the different lineages within the classes Bacilli and γ-proteobacteria still showed considerable variation in their PGM profiles, as depicted by the shading in Fig. 2 and Fig. S1. For example, of the 3 species within the family Alteromonadaceae (γ-proteobacteria), one contains dPGM, another contains iPGM and the third contains both. Variation also existed even at the species level: of two species of *Pseudoalteromonas* (γ-proteobacteria), one contains iPGM while the other has both dPGM and iPGM (Fig. S1, Table S1). Other classes of bacteria such as the Clostridia and α-proteobacteria showed yet more variation in their PGM profiles (Figs 3, 4). All 19 *Clostridium* spp. genomes contain iPGM but 3 of these additionally contain dPGM. Similarly, amongst the 7 genomes within the order Thermoanaerobacterales (Clostridia) examples exist of those containing just dPGM or iPGM or both. All 3 species of *Thermoanaerobacter* contain dPGM but 2 of them also have iPGM (Fig. 3, Table S1). The order Rhizobiales (α-proteobacteria) has a particularly haphazard PGM distribution with individual species in 2 genera (*Bradyrhizobium* and *Methylobacterium*) showing variable PGM profiles. However, the iPGM identified in *Bradyrhizobium* sp. BTAi1 consists of only the N-terminal 225 amino acids and is followed by a transposase so we considered it a pseudogene. Of the 6 sequenced strains of *Rhodopseudomonas palustris,* 4 contain only iPGM while the remaining 2 have only dPGM (Fig. 4, Table S1). Strains of this species are known to have variable gene contents and the two strains that contain only dPGM are more similar to each other than to the other isolates [30]. Other classes of bacteria showed variable levels of PGM heterogeneity (Tables 1, S1). Of 53 Actinobacteria genomes all but 2 contain solely dPGM. However, *Rubrobacter xylanophilus* contains iPGM of archaeal origin as its only PGM, while *Streptomyces coelicolor* has both bacterial iPGM and dPGM. The sister species, *S. avermitilis and S. griseus*, have only dPGM. Within the δ-proteobacteria, a similar species-level variability was observed in the genus *Geobacter* where all 5 sequenced genomes encode both bacterial and archaeal iPGM, but 3 genomes additionally contain dPGM. A further interesting example of PGM diversity was seen between the two Candidatus *Phytoplasma* spp. (Mollicutes). Candidatus *P. australiense* has iPGM and an intact glycolytic pathway, whereas Candidatus *P. mali* has an incomplete glycolytic pathway that terminates in glyceraldehyde-3-phosphate and consequently lacks any form of PGM.

### Bacteria encoding more than one dPGM protein

As mentioned above, 34 genomes contained more than one dPGM gene, and frequently members of the same genus differed in this respect. For example, *Bacteroides thetaiotaomicron* and *B. vulgatus* (Bacteroidetes) each contain 2 dPGM genes, while the different strains of *B. fragilis* have only one (Table S1). Similar numerical dPGM variations exist between different species of *Methylobacterium* and *Rhizobium* (both α-proteobacteria), and between different strains of *Frankia* (Actinobacteria) and *Bacillus cereus* (Bacilli) (Table S1). In the case of *Rhizobium* spp, the two sequenced strains of *R. etli* each have 2 dPGM genes, while *R. leguminosarum* has one. In each of the *R. etli* genomes, the additional dPGM sequence is encoded by one of the extrachromosomal plasmids. Although *R. leguminosarum* contains 6 plasmids none encodes a second dPGM. Most species of *Burkholderia* (β-proteobacteria) have 2 or 3 chromosomes with or without additional plasmids. We determined that of the 21 sequenced species or strains, only *B. xenovorans* has 2 dPGM genes and that one copy is located on a plasmid. Other species also have their different dPGM genes encoded by different molecules. For example, *Cyanothecae* sp. (Chroococcales) has both a circular and linear chromosome plus 4 plasmids and each of the chromosomes encodes dPGM. Similarly, the α-proteobacterium, *Phenylobacterium zucineum*, has 3 dPGM genes, one located on the chromosome and two on the single large plasmid. The presence of 2 or more dPGM genes appeared to correlate with larger genome sizes since no occurrence of duplicate dPGM genes was found in the smallest bacterial genomes (about 20% of all genomes). The smallest genomes with 2 dPGM genes were those found in the order Lactobacillales (smallest genome ∼1.8 Mb). Excluding these, all remaining examples were over ∼3.7 Mb and occurred in the top 45% of genomes ranked by size (Table S1). This observation is consistent with previous data correlating greater numbers of paralogous protein families with larger genome sizes [32].

### Lateral Gene Transfers

We reasoned that the patchy phyletic profiles of dPGM and iPGM we observed across the bacterial domain could be partly attributable to LGTs. However, inference of LGT events based on similarity search analysis has several limitations [33], [34]. A combination of methods such as BLAST search, phylogenetic tree construction, nucleotide composition comparisons and gene distribution pattern analyses generally provide more robust predictions of LGTs. However, phenomena including gene loss, differing evolutionary rates, convergence, selection, mutation and polymorphisms plague all these methods to various extents [33]. For large data sets similarity searches still provide a reasonable and quick indication of LGT events.

#### Examination of genomes with two or more predicted iPGM genes

Initially we examined genomes with two or more copies of either PGM form to highlight likely occurrences of LGT. Therefore we examined in detail the duplicate iPGMs identified by our bacterial iPGM queries in only 4 of the 702 genomes (described above). One of the 2 iPGMs of *Acidithiobacillus ferrooxidans* matched closely to related γ-proteobacteria while the second copy had only one γ-proteobacterial hit (other than to itself) among the 20 best hits, representing 14 different genera. These top hits for this second dPGM had comparable BLAST bit scores and were almost exclusively to certain members of the order Clostridiales and to δ-proteobacteria but included the archaeal organism, *Methanosaeta thermophila*. We observed that the PGMs from these Clostridial, δ-protoebacterial and Methanosarcinale organisms, many of which are thermophilic, frequently grouped together in our TBLASTN outputs indicating their sequence similarity, as noted previously [15], [35]. Many archaea belonging to the order Methanosarcinales are found in fresh water and marine sediments so it is perhaps not surprising to find genes shared with anaerobic soil bacteria such as *Clostridium* spp. Indeed, one-third of the ORFs from *Methanosarcina mazei*, including a predicted iPGM, have their closest homolog in the bacterial domain, indicative of widespread LGT events [35]. Thus it appears that one iPGM copy in *A. ferrooxidans* may be the result of an ancient LGT. Of the two iPGM copies in the δ-proteobacterium *Sorangium cellulosum*, one shared greatest similarity with other δ-proteobacteria, Clostridiales and other proteobacterial groups. However, the second copy had greatest similarity with a very restricted set of bacteria (3 other δ-proteobacterial species, 1 γ-proteobacterium and 3 species of the spirochaete *Leptospira*), but was otherwise most similar to kinetoplastid protozoans and plants. The phylogenetic relatedness of plants and kinetoplastids is known and many kinetoplastid proteins, including iPGM, are believed to have a plant or cyanobacterial origin [36], [37]. However, the *S. cellulosum* gene had little similarity to any extant sequenced cyanobacterium. Interestingly, the trypanosomatid glycolytic enzymes, phosphofructokinase and glyceraldehyde phosphate dehydrogenase, appear to have spirochaete origins leading to the suggestion that various trypanosomatid housekeeping genes may have been acquired by an ancestral LGT from spirochaetes [36]. It is likely that the second iPGM copy we detected in *S. cellulosum* is also the result of an LGT from a spirochaete although the possibility of an interdomain LGT from eukaryotes is not ruled out. We determined that one iPGM copy in *Pseudomonas putida* F1 contained an in-frame stop codon and should therefore be considered a pseudogene. This finding makes the *P. putida* F1 strain similar to other sequenced strains in having just one full-length iPGM open reading frame. The two iPGM copies in the Clostridial bacterium *Desulfotomaculatum reducens* appeared to be the result of a gene duplication, with the predicted proteins sharing 90% similarity and generating almost identical TBLASTN results. Therefore, of the four instances of two “bacterial-like” iPGMs in one bacterial genome, one is explained by a pseudogene, one represents probable gene duplication while two appear to be the result of LGT.

#### Examination of genomes with two or more predicted dPGM genes or phylogenetically aberrant PGM profiles

We also examined genomes with unusual PGM composition in comparison to closely related species, and genomes with two or more dPGM genes, for candidate LGT events. As mentioned above, of 53 Actinobacteria genomes, *Streptomyces coelicolor* was the only species that contained bacterial-like iPGM. This protein had similarity to a variety of other bacterial groups but predominantly to proteins from cyanobacteria, fimicutes and δ-proteobacteria, indicating a likely LGT event. Similarly, the dPGM of *Pseudoalteromonas atlantica* (γ-proteobacterium) had greatest similarity to proteins from the Chroococcales, Chlamydiae and plants as well as to a single member of the Aquificae. The ancient ancestral relationship of cyanobacteria (eg. Chroococcales), Chlamydiaceae and plant chloroplasts is known [38], but the unusual finding of a gene with high similarity to members of these groups within the γ-proteobacteria is suggestive of a LGT. We found that the TBLASTN results for one dPGM protein from those species having more than one dPGM gene, or that have dPGM when closely related species do not, were often broadly similar. For example, one dPGM protein from the β-proteobacterium *Nitrosomonas europaea* had similarity to dPGM proteins from *Janthinobacterium* sp., *Herminiimonas arsenicoxydans* (both β-proteobacteria with two dPGM genes) and to only the 3 species of *Geobacter* (δ-proteobacterium) that contain dPGM in addition to iPGM. We also observed that many of the highest-ranking hits from these various dPGM queries were to members of the Chlorobia, suggestive of either a shared ancestry or LGT events. Many of these bacterial dPGM queries also showed similarity to dPGMs from lower eukaryotes, notably the slime mold *Dictyostelium discoideum*, the hydrozoan *Hydra magnipapillata*, and the protozoan *Trichomonas vaginalis*. In many cases (eg. *Burkholderia xenovorans*, *Nitrosomonas europaea*, *Geobacter* spp.), the hits to these eukaryotic dPGMs were amongst the top 6 BLAST hits. We analyzed these eukaryotic proteins in more detail and determined that in all cases their own top BLAST hits were to bacteria (Chlorobia members in the cases of *T. vaginalis* and *D. discoideum*; β-proteobacteria in the case of *H. magnipapillata*). Interestingly, *T. vaginalis* also contains iPGM and clustering of this protein with bacterial iPGM has been noted while other protozoans with iPGM formed a monophyletic group [39]. Other inter-domain LGTs have been described or implicated previously for PGM [15], [35], [37], [40].

#### Archaeal type PGMs in bacterial genomes

We found no evidence of archaeal type dPGM genes in bacteria. The 43 bacterial genomes that contained the 50 archaeal type iPGM genes were not randomly distributed throughout the bacterial domain. Classes such as the Deinococci, Aquificae and Thermotogae that contain predominantly or exclusively thermophilic species accounted for many of the archaeal type iPGMs (Tables 1, S1). With the exception of *Deinococcus radiodurans* and 3 *Dehalococcoides* spp., all 18 bacteria with archaeal iPGM as their only PGM form are thermophilic. Of the bacterial orders with larger numbers of sequenced genomes, only the Bacteroidetes, Clostridia and δ-proteobacteria had representatives with archaeal type iPGM, and even within these groups, some species such as *Clostridium thermocellum* and *Pelotomaculum thermopropionicum* are thermophiles. Genome analyses have previously indicated massive gene exchange between thermophilic bacteria and archaea [41], [42] with as much as 25% of the bacterial proteome being most similar to archaeal proteins.

Of 19 *Clostridia* spp., only 3 had archaeal iPGM (Table S1). The gene in *C. phytofermentans*, although similar to that from *C. thermocellum*, contains an in-frame stop codon and is considered a pseudogene. The predicted proteins of *C. themocellum* and *C. novyi* have relatively low similarity to each other and gave quite different TBLASTN results, showing highest similarity to different groups of archaea, indicative of different ancestral origins. The 3 *Dehalococcoides* spp. all have two archaeal type iPGM genes. Although comparisons between species showed that the gene pairs are very similar, comparison of the two predicted proteins in any species again points to different phylogenies. Similarly, the single archaeal iPGM in *Pelobacter propionicus* (δ-proteobacteria) is similar to one of two such genes in *P. carbinolicus*. However, the second archaeal iPGM in *P. carbonolicus* is quite divergent. The two iPGMs of *Thermodesulfovibrio yellowstonii* also appeared to have different archaeal origins. The δ-proteobacterium *Syntrophus aciditrophicus* encodes 3 archaeal type iPGMs, which share only about 45% amino acid similarity and also appear to derive from different groups of archaea.

We developed a bioinformatic approach to investigate the archaeal groups that have greatest similarity to the archaeal-like iPGMs identified in bacterial genomes. We used the 50 archaeal-like iPGM proteins as queries of all complete archaeal genome sequences that represent 48 distinct archaeal species (Table S3). We determined that overall, the archaeal iPGMs from bacterial genomes had greatest similarity with members of the phylum Euryarchaeota, most notably, in decreasing order, to the classes Methanobacteria, Methanomicrobia and Methanococci (Fig. S2). However, the highest scoring individual hits were to the Methanomicrobial species *Methanococcoides burtonii*, *Methanosarcina spp*., and *Methanosaeta thermophila*. This is consistent with the reported high similarity of iPGM from these archaea and iPGM from bacteria, and the observation that *Methanosarcina mazei* and its close relatives appear to have exchanged genetic information by LGT with the bacteria that share their environment on multiple occasions [15], [35].

### Bacterial genomes encoding both dPGM and iPGM

Both PGM forms were detected in 115 genomes (16% of total) (Fig. 1; Table 1). While an archaeal iPGM never accompanied dPGM in the absence of bacterial type iPGM, 10 genomes contain all 3 types. (Fig. 1; Table S1) With the exception of the *Clostridium phytofermentans* pseudogene (discussed above), the remaining 9 genomes were restricted to the Bacteroidetes and δ-proteobacteria. The majority of species with both bacterial type PGM NISE, but not an archaeal-type example, were found within the Bacilli and γ-proteobacteria, particularly the family Enterobacteriaceae, (Table 1), but this observation is mostly accounted for by the large numbers of sequenced genomes for genera such as *Bacillus*, *Staphylococcus*, *Escherichia*, *Salmonella*, *Klebsiella* and *Yersinia*.

In looking at the dPGM and iPGM proteins predicted by each genome that encodes both forms, we noted that frequently the dPGM had unusual BLAST matches, similar to several of the dPGM proteins encoded by genomes with two or more dPGM genes (see above). For example, within the phylum Firmicutes (Clostridia/Bacilli), all *Listeria* spp and several species of *Clostridium*, *Bacillus* and *Thermoanaerobacter* have both PGM NISE forms; their dPGM proteins showed high similarity to various members of the Chlorobia as well as to lower eukaryotes such as *D. discoideum* and *H. magnipapillata.* Notably, the dPGM protein from *Desulfovibrio desulfuricans* had best BLAST match to the dPGM from the eukaryote *H. magnipapillata* followed by various Chlorobia members rather than to other δ-proteobacteria and might represent another candidate LGT event. We observed that another subset of the dPGM proteins predicted by genomes with both NISE forms had similarity to the same restricted set of bacteria and to certain yeasts (eg *S. pombe*), and some lower eukaryotes. Closer inspection of the BLAST results for *Parvibaculum lavamentivorans*, *Methylobacterium* spp (both α-proteobacteria) and *Myxococcus xanthus* (δ-proteobacteria) for example, revealed that these similarities were at least in part accounted for by the proteins resembling the characterized *S. pombe* dPGM [23], [24] in lacking a ∼25 aa region involved in dimerization/tetramerization. This finding further supports our notion that several bacterial dPGM proteins are active as monomers.

There appeared to be a strong correlation between the presence of both PGM NISE forms and genome size (Table S1). We found that of 115 genomes encoding both dPGM and iPGM, 85 were larger than 4 Mb. In fact, only 3 such genomes were smaller than 2.5 Mb (2 *Thermoanaerobacter* spp, ∼2.4 Mb and the unclassified bacterium *Elusimicrobium minutum*, ∼1.6 Mb). These genomes were the only examples found in bottom third of the list of 702 sequenced genomes ranked by size. This correlation is similar to the one we observed linking duplicate dPGM genes with larger genomes (see above) and supports the published observation that smaller genomes encode disproportionally fewer analogous enzymes (NISE) [1], [3]. Our data indicate that the presence of PGM paralogs or both NISE forms is a feature predominantly enjoyed by bacteria with larger genomes.

### Characterization of the PGM NISE forms of *E. coli*

The co-occurrence of dPGM and iPGM in the same organism is found in diverse bacterial groups (Table 1), yet only in *E. coli* has the PGM activity of both forms been investigated [6]. Biochemical and genetic studies are ultimately necessary to verify NISE predictions made by bioinformatic means. We therefore produced recombinant *E. coli* PGM enzymes for a more detailed characterization and exploited the genetic tractability of *E. coli* to create strains deficient for each PGM protein so as to gain further insight into their cellular roles and their status as functional NISE.

### Expression and activity of *E. coli* dPGM and iPGM

Recombinant dPGM and iPGM were abundantly overexpressed in *E.coli* and subsequently purified by nickel-nitrilotriacetic acid chromatography. Imidazole (100 mM for iPGM; 200 mM for dPGM) in the elution buffer resulted in release of the proteins from the nickel resin with a high degree of purity. The yield of each protein was in excess of 300 mg per liter. The sizes of dPGM and iPGM bearing vector-encoded N-terminal T7 and C-terminal His_6_ tags were consistent with their calculated molecular masses of 31 kD and 58.6 kD, respectively (Fig. S3A and B). Both *E. coli* enzymes exhibited PGM activity as evidenced by the consumption of NADH by the coupling enzymes used in the assay (Fig. S3C). The slopes of the curves in the figure were used to calculate PGM specific activities of ∼1.8 units/mg and 229 units/mg for iPGM and dPGM, respectively. This result is in agreement with an earlier report of the significantly higher specific activity of *E. coli* dPGM compared to iPGM [6]. However, in both studies, iPGM activity was determined in buffer containing magnesium, yet manganese appears to be the preferred ion for bacterial iPGM enzymes that have been characterized (see [43] for review). Addition of 1 mM manganese to the assay buffer resulted in more than a 4-fold increase in iPGM activity (Fig. S3C) yielding a specific activity of ∼8 units/mg. Somewhat surprisingly, the activity was also enhanced when assayed in the presence of cobalt (data not shown). *Clostridium perfringens* iPGM has higher activity with cobalt than with manganese although biochemical evidence suggests that the latter ion is used *in vivo* [44]. Similarly, manganese, rather than cobalt, is likely the physiologically relevant ion for *E. coli* iPGM also since it has been found integrally bound in this enzyme [6] and is the more abundant ion in the cell [45]. Although we demonstrated that certain ions enhanced iPGM activity, the level of activity was still significantly lower than that of dPGM. This relatively low specific activity of *E. coli* iPGM may not result directly from the coexistence of dPGM since bacterial iPGM enzymes can be of low activity (∼1 unit/mg or less) even in species that lack dPGM [46], [47], [48], [49]. This is in contrast to eukaryotic iPGMs where specific activities are typically in the range of 50 to 400 units/mg [13], [50], [51]. The activity of dPGM was unaffected by the addition of manganese as expected (data not shown) since dPGM enzymes are not metalloenzymes [8]. However it was sensitive to vanadate, a known inhibitor of dPGM [52], with an IC50 of 0.65 mM (data not shown).

### Evaluation of phosphatase activity

Bioinformatic analyses originally suggested the presence of both dPGM and iPGM in *Bacillus subtilis* [6], [9], [53]. However, unlike the situation in *E. coli*, it appeared that iPGM accounted for the major PGM activity while the predicted dPGM had little or no activity [46], [54]. Further studies determined that the predicted dPGM was a broad specificity phosphatase [11], a member of the acid phosphatase superfamily to which dPGM belongs. Deletion of *B. subtilis* iPGM resulted in a severe growth phenotype and asporulation [49] while deletion of the phosphatase had no effect [54]. We explored the possibility that iPGM, the less active PGM in *E.coli*, might similarly function as a phosphatase as suggested previously [6]. However, we could not detect any phosphatase activity when the protein was assayed against the general phosphatase substrate, *p*-nitrophenyl phosphate, using buffers and metal ions (Mg^2+^, Co^2+^ or Zn^2+^) preferred by bacterial alkaline phosphatases [55]. Our alkaline phosphatase positive control, calf intestinal phosphatase, was active under all conditions tested (data not shown). The finding of manganese, rather than Mg^2+^, Co^2+^or Zn^2+^, bound to *E. coli* iPGM [6] is also consistent with its function as a PGM [43] rather than an alkaline phosphatase. We note that although both *E. coli* iPGM and dPGM function as PGMs, additional cellular functions cannot be ruled out.

### Characterization of *ΔiPGM* and *ΔdPGM* mutant strains

**Figure 5:**
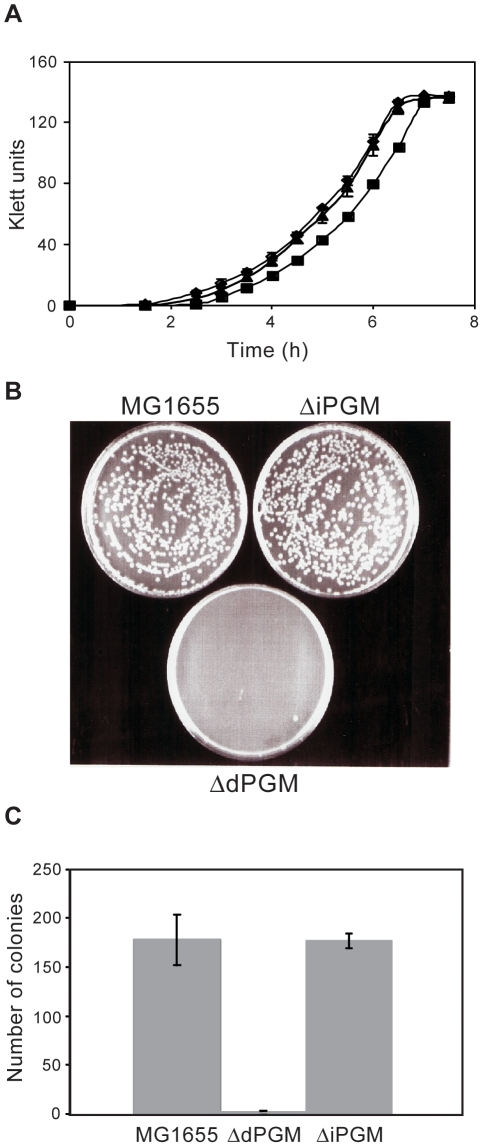
Phenotypes of *ΔdPGM* and *ΔiPGM* mutant strains. Panel A: Parental wild-type MG1655 *E. coli* (♦) and *ΔdPGM* (▪) and *ΔiPGM* (σ) mutant strains grown in minimal medium overnight were inoculated into 10 ml fresh minimal medium to give initial OD_600_ values of 0.03. Growth was monitored by determining turbidity (Klett units) during incubation at 37°C. Each data point represents the mean Klett value of triplicate cultures (± S.D.). Panels B and C: Overnight MOPS minimal medium cultures of parental wild-type MG1655 *E. coli* and the *ΔdPGM* and *ΔiPGM* mutant strains were serially diluted in minimal medium and 100 µl of each dilution plated to LB agar. Cells were grown at 37°C and the number of colonies counted. Each dilution of each strain was plated in quadruplicate. Representative plates at 1×10^−5^ dilution are shown (B) and the mean numbers of colonies (± S.D.) per plate at 1×10^−6^ dilution are plotted (C).

We prepared strains deleted for each of the predicted PGM genes in the wild-type *E. coli* K-12 strain, MG1655, using established methodology [20]. Repeated attempts to create a *ΔiPGM*, *ΔdPGM* double deletion by targeting the remaining locus in each of the mutant strains were unsuccessful. Although we did not attempt creation of the double deletion by alternative methods, we interpret this result as indicative of an absolute requirement for some form of PGM. Both mutants were healthy when grown in LB medium, but a growth lag was identified using minimal medium for *ΔdPGM*. (Fig. 5A), consistent with the higher enzyme activity of dPGM. This growth lag was seen as a delay in exiting stationary phase in the *ΔdPGM* strain relative to *ΔiPGM* and MG1655. Doubling times for both mutants and the MG1655 parent were similar during logarithmic growth in this medium. Similar results were obtained using *iPGM* and *dPGM* transposon insertion mutants (data not shown) supplied by Dr F. Blattner, University of Wisconsin. A clearer phenotype emerged when overnight cultures in minimal medium were serially diluted then plated to LB agar (Fig. 5B and C): *ΔdPGM* failed to form colonies after 24 h growth. Colonies appeared only between 48 and 72 hrs. This phenotype of *ΔdPGM* on solid medium confirms that observed in liquid culture, suggesting a general problem in exiting stationary phase in *ΔdPGM* cells. In contrast, when logarithmic phase cultures were diluted and plated on solid medium, colony formation was normal (data not shown). During stationary phase, energy metabolism is limited and primarily consists of pathways that scavenge potential nutrients from the medium and from within the cell [56]. However, upon a return to low density in glucose-containing medium the pathways of central metabolism need to be upregulated to permit rapid growth. This lag phase during which the cell adjusts to the new conditions is extended in *ΔdPGM* cells, presumably because they also have to compensate for the absence of the major PGM activity in their glycolytic pathway. No phenotype was observed for the *ΔiPGM* mutant strain in these studies. It is possible that growth of the mutant strains in the presence of alternative carbon sources could reveal a phenotype for the *ΔiPGM* strain. However, our main goal was to develop a system to examine whether the two PGM enzyme forms do indeed have overlapping functional roles within *E. coli*. This growth phenotype in *E.coli* lacking dPGM is consistent with essentiality of PGM in *Pseudomonas syringae*, *Bacillus subtilis*, *Francisella novicida* and *Mycoplasma genitalium* [49], [57], [58], [59]. Studies of PGM null mutants or gene transcript reduction by RNAi in eukaryotes such as yeast, protozoa and nematodes lend further support to the essentiality of PGM in these organisms [13], [60], [61].

### Complementation of *ΔdPGM* by dPGM and iPGM

**Figure 6:**
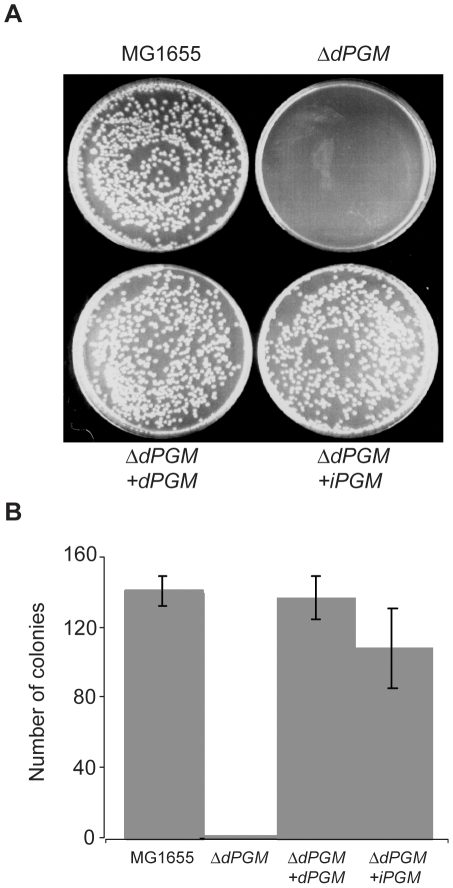
Complementation of the *ΔdPGM* phenotype by *dPGM* and *iPGM*. Overnight minimal medium cultures of parental wild-type MG1655 *E. coli*, the *ΔdPGM* mutant, and the *ΔdPGM* mutant carrying either the plasmid pKK*iPGM* or pKK*dPGM* were serially diluted in MOPS minimal medium. Triplicate aliquots of 100 µl of each dilution were plated to LB agar and the number of colonies counted after incubation at 37°C. Strains harboring plasmid constructs were grown in the presence of 100 µg/ml ampicillin. Representative plates at 1×10^−6^ dilution are shown (A) and the mean number of colonies per plate (± S.D.) are plotted (B).

The observed colony delay phenotype of *ΔdPGM* provided a system for complementation experiments using expression constructs carrying heterologous *PGM* genes. Plasmids pKK*iPGM* and pKK*dPGM* were introduced into the *ΔdPGM* strain and plated on LB agar after overnight growth in MOPS minimal medium. Strains MG1655, *ΔdPGM* and *ΔdPGM* harboring empty plasmid (pKK) were grown in parallel. The observed *ΔdPGM* growth phenotype could be restored to wild type by dPGM expressed from the plasmid pKK*dPGM* as expected. Interestingly, plasmid pKK*iPGM* also complemented the *ΔdPGM* deletion. Both expression constructs, pKK*iPGM* and pKK*dPGM*, complemented the *ΔdPGM* mutation such that the colony formation at 24 hr was similar to the parental MG1655 (Fig. 6). No colonies were evident when *ΔdPGM* was transformed with the empty vector, pKK (data not shown). These results indicate that while expression of the chromosomal copy of *iPGM* alone is not sufficient to fully compensate for the lack of dPGM activity in the *ΔdPGM* mutant, the expression of additional iPGM from a medium copy plasmid can restore the mutant cells to normal growth characteristics. It further confirms that iPGM and dPGM can function in the same metabolic pathways. Our biochemical and genetic evidence unequivocally establishing dPGM and iPGM as analogous enzymes (NISE) in *E. coli* is likely applicable to other bacteria that also encode both forms. We determined that generally such bacteria have genomes in excess of 4Mb and can presumably accommodate this apparent metabolic redundancy.

Since mammalian genomes encode only dPGM while many pathogenic bacteria, fungi, protozoans and nematodes use only iPGM, the latter has been proposed as a candidate drug target for novel treatments for various infectious diseases [6], [13], [25], [47]. The development of null mutants of both dPGM and iPGM in *E. coli* makes possible a whole organism screen for identification of potential inhibitors with specificity for iPGM. Similarly, compounds identified in high throughput screens against any recombinant iPGM can now be tested for specificity in a well-characterized bacterial system.

### Concluding remarks

The widespread occurrence of NISE is becoming increasingly apparent as more genome sequences are reported [1], [3], [62], [63], [64]. The phenomenon is attracting attention not only from an evolutionary perspective, but also because of its confounding implications for accurate genome annotation and metabolic pathway reconstruction, and for its potential in highlighting drug targeting opportunities against various pathogenic organisms [1], [3], [4], [64], [65], [66], [67]. For example, a web-based tool AnEnPi (Analogous Enzyme Pipeline) has been developed that enables researchers to identify NISE in pathogen and host genomes [64]. Since vertebrates only contain dPGM [10], the iPGM protein of any pathogen encoding only that form represents a candidate drug target. In our analysis, we identified 243 bacterial genomes (∼35% of genomes examined) that encode only iPGM. These include pathogenic representatives from a variety of genera such as *Mycoplasma*, *Campylobacter*, *Coxiella*, *Vibrio*, *Helicobacter*, *Pseudomonas*, *Leptospira*, *Legionella* amongst others. Thus iPGM represents a potential drug target in diverse bacterial groups.

Glycolysis is an essential component of central metabolism and is conserved in almost all prokaryotes and eukaryotes. However, several glycolytic enzymes such as PGM, phosphofructokinase, and lactate dehydrogenase have truly analogous forms (NISE), while others such as glucokinase, aldolase, FBPase and phosphoglucoisomerase, have highly variant, albeit structurally similar, forms [3], [68]. These enzymes, encoded by multiple gene sequences, almost exclusively function in the early stages of glycolysis or in associated areas of hexose metabolism. PGM is unusual since it is the only variant enzyme found in the so-called trunk pathway from glyceraldehyde-3-phosphate to pyruvate which is otherwise highly conserved and indicative that the ancestral function of the glycolytic pathway was biosynthetic rather than glycolytic [3], [68].

*E. coli* dPGM and iPGM have no sequence or structural similarities and use dissimilar catalytic mechanisms. Their PGM activities, shown both in this study and previously [6], coupled with our mutant analyses demonstrating overlapping and supplementary functions in the cell unequivocally establish the two forms as NISE. Furthermore, enhancement of iPGM activity by manganese agrees with earlier data reporting this ion bound in the *E. coli* enzyme [6], and supports the lack of phosphatase activity we report since known alkaline phosphatases require ions other than manganese. Although our experimental data derives from the model organism, *E. coli*, we anticipate it is valid for diverse bacteria that contain both predicted PGM forms. Our finding that bacteria that encode both PGM NISE predominantly have larger genomes is consistent with their individual functions being supplementary. Presumably smaller, compact genomes are less able to accommodate and maintain genes encoding functionally equivalent proteins.

The presence of both PGM NISE forms in the same organism is found in diverse bacterial groups (Table 1), but is particularly prevalent in the Bacilli and Enterobacteriaceae (γ-proteobacteria). In most bacterial taxa that have several representative sequenced genomes, the PGM profile is non-uniform. Different genomes may have both forms, as *E. coli* does, or only dPGM or iPGM, or, in a few cases, neither. Further complexity results from the presence of two or more bacterial-type dPGM genes in many genomes and from the occurrence of archaeal iPGM in over 40 genomes (Table 1). The patchy distribution of the NISE forms appears to be partly due to LGT but is undoubtedly due to gene losses in specific lineages. The PGM NISE forms are a clear case of a phenomenon coined non-orthologous gene displacement [69], [70]. In its simplest form this is represented by the presence of non-orthologous genes that encode enzymes capable of carrying out the same reaction being present in an ancestral genome followed by lineage-specific gene losses [69]. Non-orthologous gene displacement could also follow events such as LGT or enzyme recruitment that lead to the presence of both NISE forms in the same genome [71], again followed by losses in some lineages. Such mechanisms seem most likely in the case of PGM. Firstly, both iPGM and dPGM are members of the larger alkaline phosphatase and acid phosphatase enzyme superfamilies, respectively [7], [8], [9] and could evolve by recruitment by shifting the substrate specificity of a related but different enzyme. Secondly, PGM genes appear to have moved frequently between different bacteria and between the domains of life (this study, and [15], [35], [37], [40]) and introduced new PGM coding potential into recipient genomes. Regardless of the origins of the two enzyme forms, functional redundancy, where a bacterium contains both NISE, must be a prerequsite for non-orthologous gene displacement, where essential genes are concerned, and must precede any subsequent selective loss of one gene. Whether any of the bacteria we report as containing both PGM NISE are undergoing the early stages of non-orthologous PGM gene displacement or reflect a retained ancestral condition is an open question. Nonetheless, it appears that enzyme recruitment, gene duplications, gene losses, LGT events and non-orthologous gene displacement have contributed to the intriguing non-uniform distribution of analogous PGM enzymes (NISE) across the bacterial domain that we see today.

## Supporting Information

Figure S1 Distribution of PGM types across 184 completed genome sequences from the Class γ-proteobactria. Taxonomic nodes (left to right) are Class, Order, Family, Genus (or species in the case of 3 incompletely classified bacteria at the bottom of the Figure). Taxa with genomes containing only iPGM are shaded yellow, those with only dPGM are shaded blue, those with both iPGM and dPGM are shaded green, while taxa with non-uniform PGM profiles are shaded pink. Taxa with no PGM are unshaded. The numbers in boxes accompanying each taxon identifier correspond to (left to right) number of genomes with only dPGM, only iPGM, both dPGM and iPGM, and no PGM.
(0.05 MB PDF)

Figure S2 Average best TBLASTN bit score of archaeal type iPGM sequences from bacterial genomes queried against completed archaeal genomes. The 50 archaeal type sequences identified in 43 bacterial genomes were compared to the completed genomes of 48 archaeal species. The identities of the archaeal species, numbered 1 to 48 on the y-axis, are provided in Table S3. Different phyla within the kingdom archaea are differentially shaded. Classes of archaea having multiple representative genome sequences are indicated above the shaded boxes along with the average bit score for that entire class.
(0.02 MB PDF)

Figure S3 Overexpression and purification (Panels A, B) and activity (Panel C) of recombinant dPGM and iPGM. Panels A (dPGM) and B (iPGM): Lanes: 1, *E. coli* total protein without induction with IPTG; 2, *E.coli* total protein following induction with IPTG; 3, soluble *E. coli* proteins after cell disruption; 4, flow-through from the nickel column; 5, Wash of nickel column prior to elution; 6 and 7, elution fractions from nickel column using imidazole (200 mM for dPGM, 100 mM for iPGM). Panel C: PGM activity of recombinant dPGM and iPGM. Conversion of 3-PG to 2-PG by 0.25 µg dPGM (▪) and 10 µg iPGM (▴) assayed in standard, magnesium-containing buffer. Conversion of 3-PG to 2-PG by iPGM in buffer supplemented with 1 mM manganese chloride is shown for comparison (*). A control lacking any recombinant protein is also shown (♦). Conversion of 3-PG to 2-PG is determined indirectly by a decrease in NADH concentration as measured by its absorbance at 340 nm. Consumption of NADH is directly proportional to PGM activity.
(8.63 MB TIF)

Table S1 Identification of orthologs of iPGM, dPGM and related superfamily member proteins across 702 complete bacterial genomes. The protein queries are as described in Materials and Methods. The number of predicted proteins in each genome sequence that match the query protein above a TBLASTN bit score of 100 are provided. Based on these hits, each genome is assigned a status of either “I” (iPGM) “D” (dPGM), “D+I” (dPGM plus iPGM) or “None” (No PGM). The assignments were made automatically or manually as described in Materials and Methods. A plus sign (+) in the column headed “Multiple Molecules” indicates that the queried genome has more than one molecule and that the multiple hits are to different chromosomes or extrachromosomal plasmids. [However, *Corynebacterium glutamicum* ATCC 13032 and *Bacillus licheniformis* ATCC 14580 have been sequenced twice resulting in duplicate molecules and are also marked “+”; *Burkholderia multivorans* ATCC 17616, also sequenced twice, has 3 chromosomes and one plasmid and has 2 dPGM hits on chromosome 1 and is marked “+”. Similarly, *Ehrlichia ruminantium* str. Welgevonden has been sequenced twice (+) and has one iPGM hit on each sequence. However, the assignment of different origins in the *E. ruminantium* genome sequences results in the hits not overlapping so being scored twice].
(0.32 MB XLS)

Table S2 PCR primers for amplification of *E. coli* dPGM and iPGM.
(1.29 MB DOC)

Table S3 The 48 archaeal species with complete genome sequences that served as subjects for TBLASTN analysis. The archaeal genomes were queried using the 50 archaeal type iPGM sequences identified in the completed bacterial genomes. The number accompanying each species corresponds to the y-axis numbering in Fig. S2. The multiple GI numbers associated with each species represent genome sequences of different strains and/or multiple molecules (eg. plasmids) of certain species/strains.
(0.03 MB DOC)
