# Supplementary figures and images for: Evolution of Bacterial Phosphoglycerate Mutases: Non-Homologous Isofunctional Enzymes Undergoing Gene Losses, Gains and Lateral Transfers
Source: PLoS One. 2010 Oct 26;5(10):e13576. doi: 10.1371/journal.pone.0013576 (PMC2964296; doi:10.1371/journal.pone.0013576)

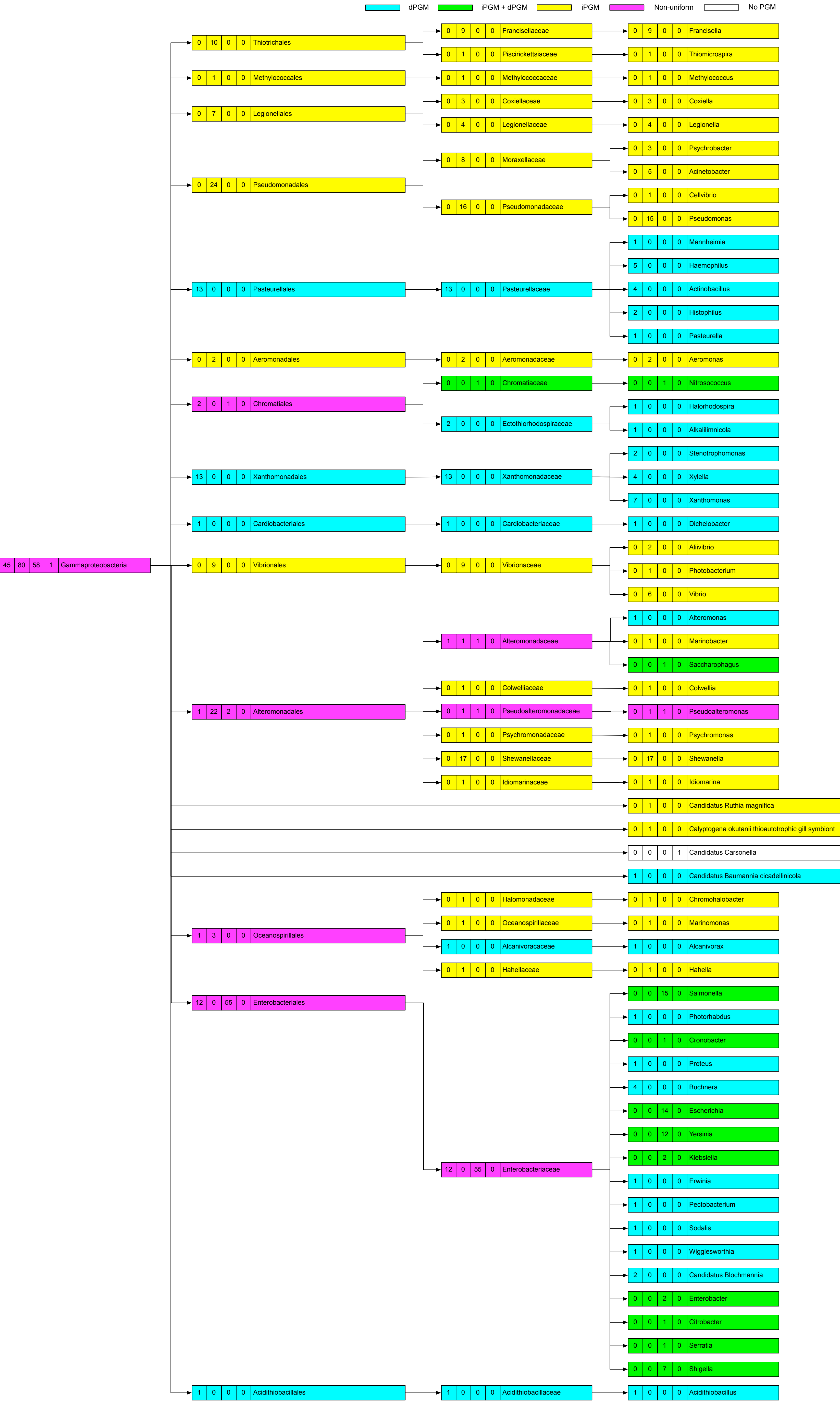

Supplement: Figure S1 — Distribution of PGM types across 184 completed genome sequences from the Class γ-proteobactria. Taxonomic nodes (left to right) are Class, Order, Family, Genus (or species in the case of 3 incompletely classified bacteria at the bottom of the Figure). Taxa with genomes containing only iPGM are shaded yellow, those with only dPGM are shaded blue, those with both iPGM and dPGM are shaded green, while taxa with non-uniform PGM profiles are shaded pink. Taxa with no PGM are unshaded. The numbers in boxes accompanying each taxon identifier correspond to (left to right) number of genomes with only dPGM, only iPGM, both dPGM and iPGM, and no PGM. (0.05 MB PDF) [file pone.0013576.s001.pdf]

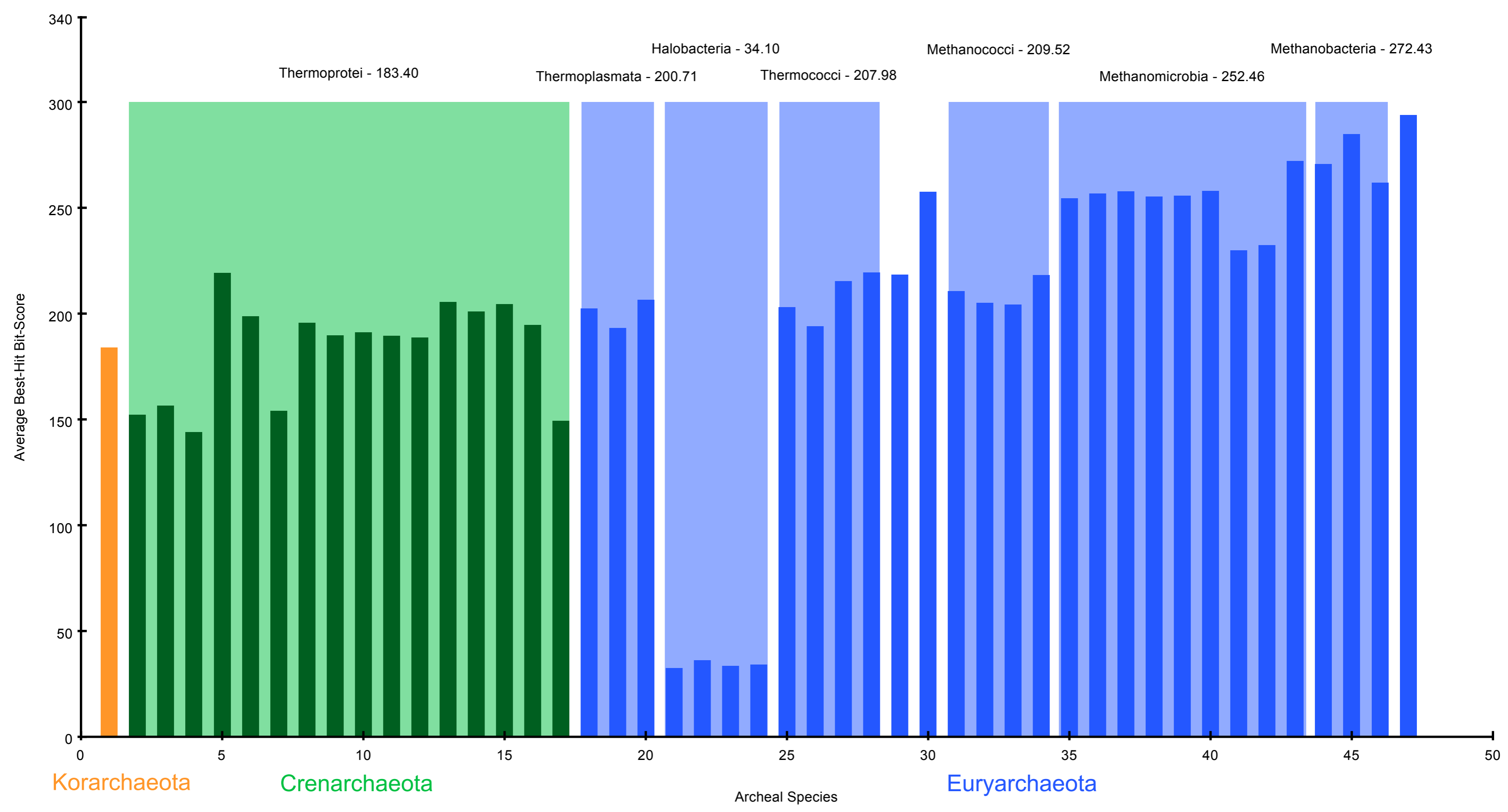

Supplement: Figure S2 — Average best TBLASTN bit score of archaeal type iPGM sequences from bacterial genomes queried against completed archaeal genomes. The 50 archaeal type sequences identified in 43 bacterial genomes were compared to the completed genomes of 48 archaeal species. The identities of the archaeal species, numbered 1 to 48 on the y-axis, are provided in Table S3. Different phyla within the kingdom archaea are differentially shaded. Classes of archaea having multiple representative genome sequences are indicated above the shaded boxes along with the average bit score for that entire class. (0.02 MB PDF) [file pone.0013576.s002.pdf]

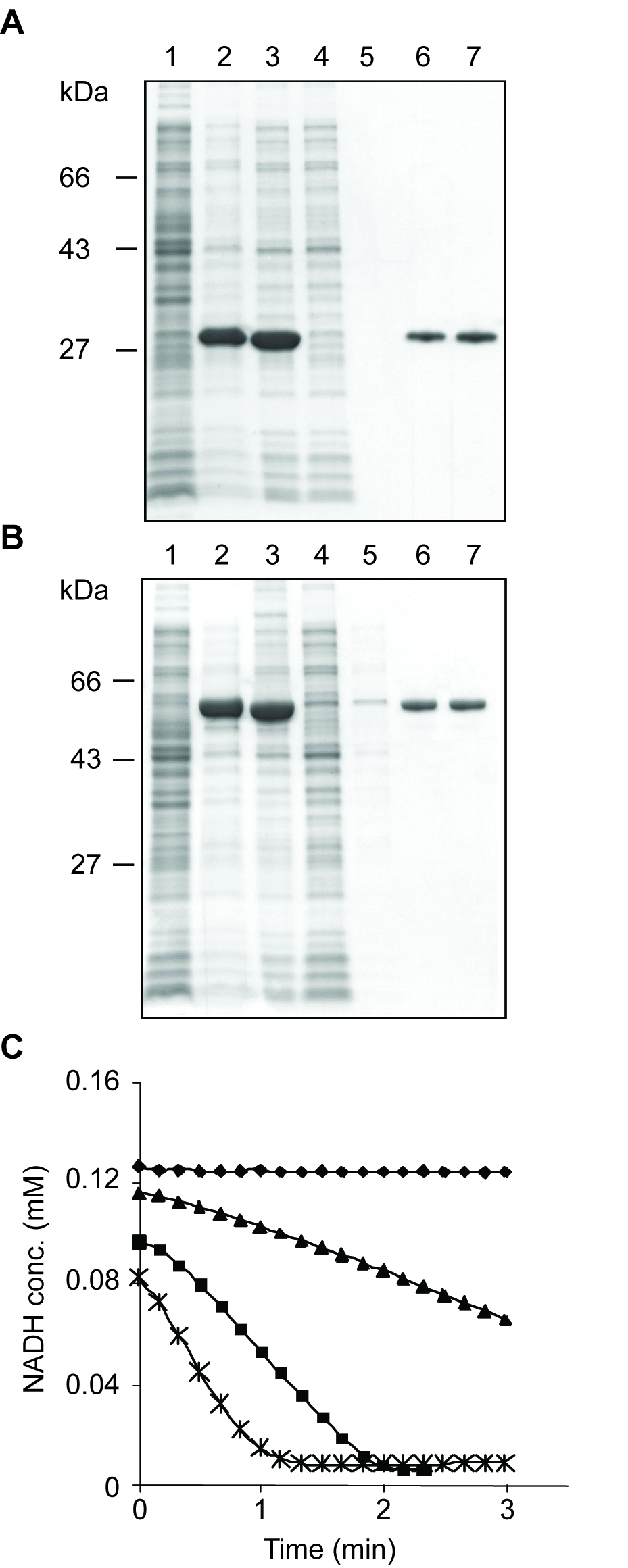

Supplement: Figure S3 — Overexpression and purification (Panels A, B) and activity (Panel C) of recombinant dPGM and iPGM. Panels A (dPGM) and B (iPGM): Lanes: 1, E. coli total protein without induction with IPTG; 2, E.coli total protein following induction with IPTG; 3, soluble E. coli proteins after cell disruption; 4, flow-through from the nickel column; 5, Wash of nickel column prior to elution; 6 and 7, elution fractions from nickel column using imidazole (200 mM for dPGM, 100 mM for iPGM). Panel C: PGM activity of recombinant dPGM and iPGM. Conversion of 3-PG to 2-PG by 0.25 µg dPGM (▪) and 10 µg iPGM (▴) assayed in standard, magnesium-containing buffer. Conversion of 3-PG to 2-PG by iPGM in buffer supplemented with 1 mM manganese chloride is shown for comparison (*). A control lacking any recombinant protein is also shown (♦). Conversion of 3-PG to 2-PG is determined indirectly by a decrease in NADH concentration as measured by its absorbance at 340 nm. Consumption of NADH is directly proportional to PGM activity. (8.63 MB TIF) [file pone.0013576.s003.tif]
