# Supplementary material for: Evolution of Bacterial Phosphoglycerate Mutases: Non-Homologous Isofunctional Enzymes Undergoing Gene Losses, Gains and Lateral Transfers
Source: PLoS One. 2010 Oct 26;5(10):e13576. doi: 10.1371/journal.pone.0013576 (PMC2964296; doi:10.1371/journal.pone.0013576)
Supplement: Table S3 — The 48 archaeal species with complete genome sequences that served as subjects for TBLASTN analysis. The archaeal genomes were queried using the 50 archaeal type iPGM sequences identified in the completed bacterial genomes. The number accompanying each species corresponds to the y-axis numbering in Fig. S2. The multiple GI numbers associated with each species represent genome sequences of different strains and/or multiple molecules (eg. plasmids) of certain species/strains. (0.03 MB DOC) [file pone.0013576.s006.doc]

Candidatus Korarchaeum

1. 170289627 Candidatus Korarchaeum cryptofilum

Thermoprotei

2. 124026906 Hyperthermus butylicus

3. 118430835 Aeropyrum pernix

4. 126464913 Staphylothermus marinus

5. 156936795 Ignicoccus hospitalis

6. 119709757,119718918 Thermofilum pendens

7. 159040592 Caldivirga maquilingensis

8. 119871520 Pyrobaculum islandicum

9. 18311643 Pyrobaculum aerophilum

10. 145590267 Pyrobaculum arsenaticum

11. 126458628 Pyrobaculum calidifontis

12. 171184485 Thermoproteus neutrophilus

13. 24473558 Sulfolobus tokodaii

14. 70605853 Sulfolobus acidocaldarius

15. 15896971 Sulfolobus solfataricus

16. 146302785 Metallosphaera sedula

17. 161527512 Nitrosopumilus maritimus

Thermoplasmata

18. 48477072 Picrophilus torridus

19. 16081186 Thermoplasma acidophilum

20. 13540831 Thermoplasma volcanium

Halobacteria

21. 76800655,76803317,76803367 Natronomonas pharaonis

22. 15789340,10803547,16119979,169235049,169237160,

16923731,169237353,169237602 Halobacterium salinarum

23. 55376107,55376144,55376187,55376228,55376280,55376412,

55376579,55376942,55380074 Haloarcula marismortui

24. 110666976,109644367 Haloquadratum walsbyi

Thermococci

25. 14518450,10954552 Pyrococcus abyssi

26. 18976372 Pyrococcus furiosus

27. 14589963 Pyrococcus horikoshii

28. 57639935 Thermococcus kodakarensis

Methanopyri

29. 20093440 Methanopyrus kandleri

Archaeoglobi

30. 11497621 Archaeoglobus fulgidus

Methanococci

31. 150400439 Methanococcus aeolicus

32. 45357563,134045046,134046898,150401930,159904396 Methanococcus maripaludis

33. 150398760 Methanococcus vannielii

34. 15668172,10954532,10954488 Methanocaldococcus jannaschii

Methanomicrobia

35. 116753325 Methanosaeta thermophila

36. 20088899 Methanosarcina acetivorans

37. 73667559,73663826 Methanosarcina barkeri

38. 21226102 Methanosarcina mazei

39. 91772082 Methanococcoides burtonii

40. 124484829 Methanocorpusculum labreanum

41. 154149549 Candidatus Methanoregula

42. 88601322 Methanospirillum hungatei

43. 126177952 Methanoculleus marisnigri

Methanobacteria

44. 84488831 Methanosphaera stadtmanae

45. 15678031 Methanothermobacter thermautotrophicus

46. 148642060 Methanobrevibacter smithii

Environmental Sample

47. 147918682 Uncultured methanogenic archaeon RC-I

Nanoarchaeum

48. 38349555 Nanoarchaeum equitans
