# Supplementary material for: Evolution of Bacterial Phosphoglycerate Mutases: Non-Homologous Isofunctional Enzymes Undergoing Gene Losses, Gains and Lateral Transfers
Source: PLoS One. 2010 Oct 26;5(10):e13576. doi: 10.1371/journal.pone.0013576 (PMC2964296; doi:10.1371/journal.pone.0013576)
Supplement: Supplementary file 4 [file pone.0013576.s005.doc]

PGM Expression

	Primer sequence (5' to 3'; BamHI and XhoI sites underlined)
iPGM-eFwd	ATAAGTGGATCCATGTTGGTTTCTAAAAAACC
iPGM-eRev	TAAGTTCTCGAGTTCCACGATGAACAGCG
dPGM-eFwd	ATAAGTGGATCCATGGCTGTAAGCTGGT
dPGM-eRev	TAAGTTCTCGAGCTTCGCTTTACCCTGGTTTG

PGM Gene Deletions

	Primer sequence (5' to 3'; pKD4 priming sites underlined)
iPGM-dFwd	TACGCAAATTTTGACTCTTGAGTATGAGGTTGTCCGCAGTGTAGGCTGGAGCTGCTTC
iPGM-dRev	GTCATGGTATTAATCGCCTTTCCCCTCATGGGGAGGGACATATGAATATCCTCCTTAG
dPGM-dFwd	TCACTTGCAGTCGGCTTTCTCATTTTAAACGAATGACGTGTGTAGGCTGGAGCTGCTTC
dPGM-dRev	AGAATTATTATCATTAAAAGATGATTTGAGGAGTAAGTATCATATGAATATCCTCCTTAG

PGM Complementation

	Primer sequence (5' to 3'; EcoRI sites underlined)
iPGM-cFwd	ATAAGTGAATTCATGTTGGTTTCTAAAAAACC
iPGM-cRev	TAAGTTGAATTCTTATTCCACGATGAACAGCG
dPGM-cFwd	GAACTCGAATTCCATGGCTGTAACTAAGCTGGT
dPGM-cRev	TAAGTTGAATTCTTACTTCGCTTTACCCTGGTTTG
